# Recent Developments in Molecular Dynamics Simulations of Fluorescent Membrane Probes

**DOI:** 10.3390/molecules16075437

**Published:** 2011-06-27

**Authors:** Luís M. S. Loura, J. P. Prates Ramalho

**Affiliations:** 1 Faculdade de Farmácia, Universidade de Coimbra, Pólo das Ciências da Saúde, Azinhaga de Santa Comba, 3000-548 Coimbra, Portugal; Email: lloura@ff.uc.pt; 2 Centro de Química de Coimbra, Universidade de Coimbra, Rua Larga, 3004-535 Coimbra, Portugal; 3 Centro de Química de Évora e Departamento de Química, Escola de Ciências e Tecnologia, Colégio Luís Verney, Rua Romão Ramalho 59, 7002-554 Évora, Portugal

**Keywords:** fluorescence, lipid bilayer, molecular dynamics, molecular simulation, membrane probes

## Abstract

Due to their sensitivity and versatility, the use of fluorescence techniques in membrane biophysics is widespread. Because membrane lipids are non-fluorescent, extrinsic membrane probes are widely used. However, the behaviour of these probes when inserted in the bilayer is often poorly understood, and it can be hard to distinguish between legitimate membrane properties and perturbation resulting from probe incorporation. Atomistic molecular dynamics simulations present a convenient way to address these issues and have been increasingly used in recent years in this context. This article reviews the application of molecular dynamics to the study of fluorescent membrane probes, focusing on recent work with complex design fluorophores and ordered bilayer systems.

## 1. Introduction

The understanding of the organization of biomembranes at the molecular level and the establishment of structure/function relationships of membrane-active biomolecules are questions of paramount importance in cell biophysics and biochemistry, whose study most frequently requires the use of advanced experimental methodologies [1]. In this regard, fluorescence is one of the most powerful and commonly used tools in biophysical studies of membrane structure and dynamics, as well as in physiological studies involving membrane systems [2,3]. Its advantages are manifold, and include extremely high sensitivity (evidenced in single-fluorophore imaging and fluorescence correlation spectroscopy techniques) and sub-nanosecond time resolution (enabling the study of processes occurring in as little as 10^−10^ s to as much as ~10^4^ s). Fluorescence spectroscopy is highly versatile, and different parameters allow retrieval of complementary molecular information on the system under study. Spectral and fluorescence lifetime/quantum yield variations are informative regarding the polarity and/or solvent accessibility of the fluorophore microenvironment and the extent of partition of a fluorophore-bearing molecule between the aqueous and membrane media, or between coexisting membrane phases or domains. Fluorescence quenching, depending on the underlying interaction mechanism and on the experimental design, can be used to study molecular aggregation, lateral diffusion, compartmentalization, or transverse location in the bilayer. Fluorescence polarization is used to measure the viscosity of the microenvironment and the kinetics of fluorophore rotation. Förster resonance energy transfer (FRET) is useful in many common situations, such as detection and characterization of membrane heterogeneity, determination of the transverse location of protein fluorophores, detection and quantification of protein/lipid selectivity, and characterization of protein oligomerization. Fluorescence microscopy adds spatial resolution to the canon, and, also because of the non-destructive nature of fluorescence techniques, is widely used in the study of live cells. An increasing number of laboratories all over the world are equipped with instruments capable of measuring microscopic fluorescence decays, thus combining spatial and time resolution. In particular, recent developments in multi-wavelength and polarization resolved imaging heave led to a widespread use of FRET imaging in studies of functional assemblies in cell membranes [4].

**Figure 1:**
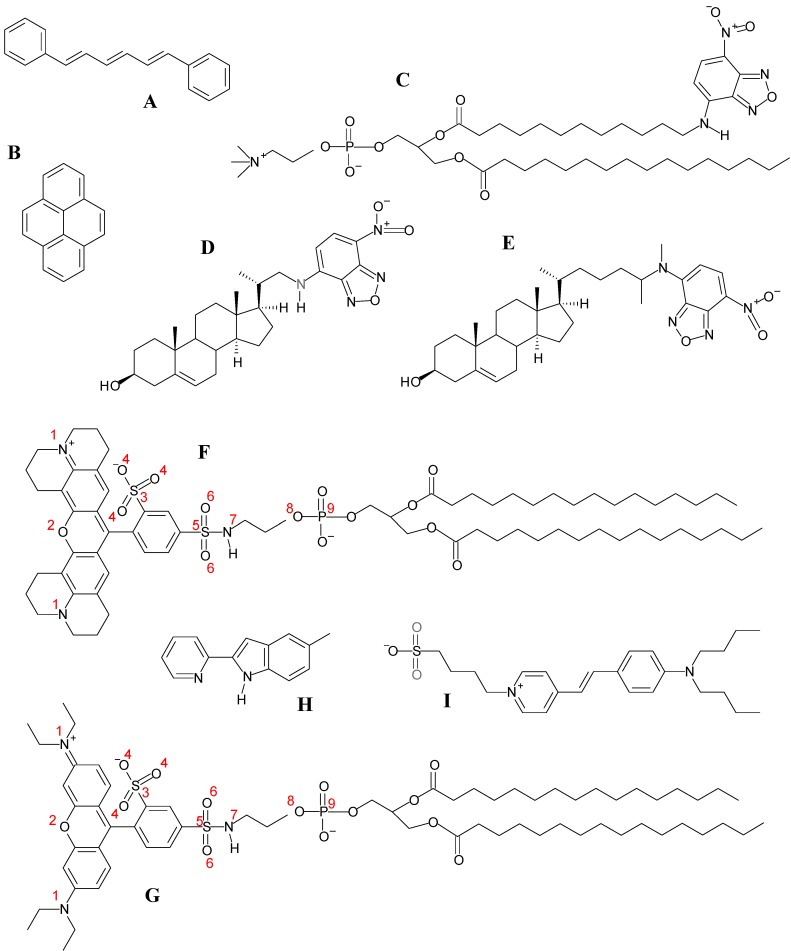
Structures of membrane probes mentioned in this article. (**A**) DPH; (**B**) pyrene; (**C**) C12-NBD-PC; (**D**) 22-NBD-cholesterol; (**E**) 25-NBD-cholesterol; (**F**) TR-DHPE; (**G**) Rhod-DPPE; (**H**) 2-(2′-pyridyl)-5-methylindole; (**I**) Di-4-ASPBS.

Because the structural unit of biological membranes is the phospholipid bilayer and phospholipids are not intrinsically fluorescent, many of the applications described above require the use of fluorescent membrane probes. Some probes bear no resemblance to lipids, but due to their hydrocarbon-based structure, are sufficiently hydrophobic to partition to the lipid environment. Examples described below are 1,6-diphenylhexatriene (DPH) or pyrene (see Figure 1A and Figure 1B for structures). Sometimes, synthetic fluorophores with useful photophysical properties are attached to aliphatic chains to increase the extent of partition. In other cases, the probes are phospholipids with a suitable fluorophore covalently attached to either the polar headgroup or one of the acyl chains, or sterols with a fluorophore label bound to either the side chain or the oxygen atom (e.g., cholesteryl esters). In all cases, the fluorescence membrane probe is a foreign molecule inserted in a host lipid matrix. Thus, perturbation of bilayer structure, dynamics of bilayer components and thermotropic behavior (that is, regarding the thermodynamics of bilayer phase transitions) is expected, and to some degree inevitable, even for low probe concentrations [5]. This raises the problem of difficulty to distinguish between legitimate membrane properties and perturbation resulting from probe incorporation. On the other hand, there is often a degree of uncertainty regarding the location of the fluorophore in the bilayer, as well as the dynamics of the bilayer-inserted probe. In this situation, the utility of the fluorescence measurement is compromised, as there is doubt regarding the bilayer region being probed, as well as whether the membrane probe used is a good mimic of the host lipid component it is supposed to emulate. In many cases, fluorescence alone is unable to solve these fundamental questions, and other methodologies, capable of independently studying the behaviors of the probe and the host lipids, are required.

In this regard, molecular dynamics (MD) simulations have been established in the past decade as a method of choice to analyze both the location and dynamics of the inserted probe and its effect on the host bilayer. A number of experimental observables (e.g., area/lipid, bilayer thickness, membrane order, electrostatic potential, lateral diffusion) can be verified in probe-free simulations, which is useful to validate the methodology. Most importantly, unique information can be obtained, regarding both probe properties and effect of probes on the host bilayer. Two years ago, we presented a review of the MD studies of fluorescent membrane probes, describing the reports then available in the literature [6]. These consisted mostly of atomistic simulations of simple nonpolar (and therefore easier to model) fluorophores in fluid disordered bilayers (easier to simulate). The studies published since then illustrate tendencies to (i) widen the range of studied probes by simulating increasingly complex fluorophores; (ii) extend simulations to ordered bilayers, e.g., containg cholesterol (which adds considerable biological relevance, given the ubiquity of the latter in the plasma membranes of mammals [1]); (iii) introduce the use of coarse-grained force-fields, which allow the study of much longer timescales and larger systems. This paper presents an updated review of this rapidly-evolving field, emphasizing these very recent trends.

## 2. Atomistic MD Simulations of Fluorophores in Lipid Bilayers

### 2.1. Apolar Fluorophores

Because of their relative structural simplicity, the first fluorescent probes simulated in lipid bilayers were small, apolar and highly symmetrical molecules, such as 1,6-diphenylhexatriene (DPH) and pyrene. DPH (Figure 1A) is a rod-shaped molecule whose fluorescence polarization is very sensitive to the microenvironment viscosity. For this reason, DPH is the archetypal membrane probe used for assessment of membrane fluidity [7,8]. Because of its hydrophobicity, DPH was traditionally expected to reside inside the apolar acyl chain region of lipid bilayers. However, its orientation in the bilayer remained an unresolved question for a long time. From analysis of time-resolved fluorescence anisotropy, Litman and co-workers proposed a bimodal angular distribution of the long molecular axis. One molecular population would have this axis aligned parallel to the membrane normal, whereas the other population would be characterized by a perpendicular alignment, compatible with a location in the bilayer midplane, between the two leaflets [9,10]. This picture was challenged by a pioneering MD simulation study, in which it was observed that the angle between the long molecular axis and the bilayer normal rarely was >60°, and no cdetected in a 72-molecule fluid 1,2-dipalmitoyl-*sn*-3-glycerophosphocholine (DPPC) bilayer [11]. However, this early work was severely restricted by the extremely short time-scale probed (≈250 ps for analysis). A more definite study, employing larger (128-molecule) DPPC fluid bilayers, simulated for 50 ns, revealed broad angular distributions of the DPH long axis, and despite a peak being observed for *f*(θ)sin(θ) (where *f* is the orientation distribution function) at θ ≈ 25°, significant distribution also occurs around θ ≈ 90° [12]. However, perpendicular orientations do not correlate with center of mass location near the bilayer center. The average center of mass transverse position was determined to lie at 0.75 nm from the bilayer center, in agreement with fluorescence quenching results [13]. Dynamic properties of DPH, such as lateral diffusion and rotational mobility, were also addressed. A later report [14] focused on the effects of DPH on the properties of the host bilayer, concluding that DPH has only a small perturbing effect on DPPC fluid bilayers (justifying the widespread use of DPH as a fluorescent membrane probe), even though significant local ordering effects are observed.

More recently, the behavior of DPH in bilayers composed of DPPC and cholesterol (with either 5 mol % or 20 mol % of the latter) was studied [15]. It is concluded that the increased order and membrane thickness resulting from the presence of cholesterol affects the location and orientation of bilayer-inserted DPH. The distance from the bilayer center to the average transverse location of DPH increases by 0.2 nm for the 20 mol % system, and the orientation distribution of the DPH long axis becomes narrower, with a single maximum for θ ≈ 10° and essentially zero values for θ ≈ 90°. Similarly to the system without cholesterol, though detectable ordering effects are observed for the DPPC acyl chains closest to the DPH molecules, no significant overall perturbations are observed in parameters such as average area/lipid and deuterium order parameters. However, for the system with 20 mol % of cholesterol, the well-known ordering effect of this component is so dominant that the additional role of DPH becomes almost negligible. This paper also focuses on a critical evaluation of the study of the orientational distribution of the fluorophore by time-resolved fluorescence anisotropy. The latter method allows the recovery of at most the first three coefficients (<*P*_0_>, <*P*_2_> and <*P*_4_>) of the Legendre polynomial series expansion of the orientational distribution function, whereas the complete function is readily available from MD simulation. It is shown that, whereas the truncation to the first three terms is still satisfactory for the more disordered systems (without cholesterol and with 5 mol %), important quantitative disagreement is observed for the cholesterol-rich ordered system.

**Figure 2:**
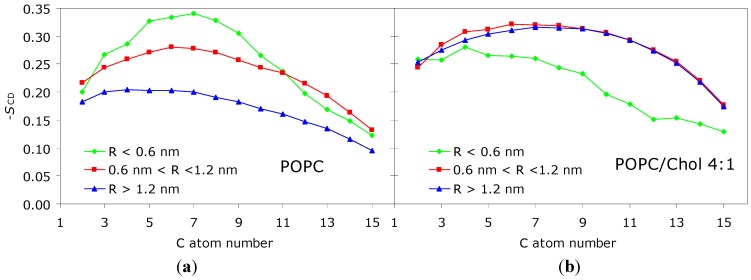
Deuterium order parameter profile of the POPC *sn*-1 chain for varying proximity to the closest pyrene molecule (*R*) in the 100-ns MD simulation of (**a**) POPC/pyrene (128:2) and (**b**) POPC/cholesterol/pyrene (120:30:2) bilayers. For *R* > 1.2 nm, the profiles are essentially identical to those obtained in absence of probe in both systems (not shown). Significant local ordering is observed for *R* < 1.2 nm in (**a**), whereas significant local disordering is observed for *R* < 0.6 nm in (**b**).

Pyrene (Figure 1B) is notable for its spectroscopic properties, including its long fluorescence lifetime (of the order of 100 ns in a variety of systems) and ability to form excimers, which prompted its common use in membrane biophysics studies [16]. Previously, pyrene has been simulated in fluid 1‑palmitoyl,2-oleoyl-*sn*-3-glycerophosphocholine (POPC; [17]) and in both fluid and gel DPPC bilayers [18]. The main conclusions of these studies may be summarized as follows: (i) pyrene has broad transverse distribution and orientation distributions, with maxima around phospholipid acyl chain carbon 5 and for perpendicular orientation of the pyrene plane relative to the bilayer plane, respectively; and (ii) overall effects of pyrene on area/lipid, lipid lateral diffusion and -*S*_CD_ are minor for the moderate probe concentrations studied. However, significant local (for *R* < 1.0 nm) effects are present. In the fluid phase simulations, acyl chain ordering is verified, similarly to DPH; in the gel phase, on the contrary, the relative number of *trans*/*gauch*e defects along the acyl chains of neighboring DPPC molecules increases, indicating a disordering effect. In agreement, pyrene incorporation causes a significant reduction and minor increase in the gel and fluid phase bilayer thickness, respectively. The behavior of phospholipid labeled with pyrene at the *sn*-2 acyl chain (1-palmitoyl-2-(1-pyrenedecanoyl)-*sn*-glycero-3-phosphocholine or PyrPC) has also been studied in fluid DPPC bilayers [19]. A local ordering effect, similar to that describe above for the free pyrene molecule, was reported. We have recently simulated pyrene in POPC/cholesterol mixed bilayers containing 20 mol % of the latter component (Loura and do Canto, unpublished data). Similarly to the effect reported by Čurdová *et al.* [18] for pyrene in DPPC gel, we observed that pyrene produces a disordering effect, increasing the area/lipid molecule and decreasing the bilayer thickness and acyl chain order parameter, and thus opposing the ordering effect of cholesterol. Although the overall variations of these parameters are modest, significant local effects are apparent, as illustrated in Figure 2 for -*S*_CD_. This observation is in contrast to that of DPH in 4:1 DPPC/cholesterol, for which a slight ordering effect was reported, as described above. The difference in behavior of the two probes is probably due to pyrene being substantially bulkier and therefore more difficult to accommodate in an ordered bilayer without inducing perturbation.

### 2.2. Polar Fluorophores

Despite having arrived later to the scene, MD simulations of design polar fluorophores are currently outnumbering those involving apolar fluorescent membrane probes. The first probes to be studied in this manner were 1-palmitoyl,2-[6-amino]dodecanoyl-*sn*-glycero-3-phosphocholine and 1-palmitoyl,2-[12-amino]dodecanoyl-*sn*-glycero-3-phosphocholine [C6-NBD-PC and C12-NBD-PC (Figure 1C), respectively] [20,21], 1,1´-dioctadecyl-3,3,3´,3´-tetramethylindocarbocyanine [DiIC_18_(3)] [22,23] and 23‑(4,4-difluoro-1,3,5,7-tetramethyl-4-bora-3a,4a-diaza-*s*-indacen-8-yl)-24-norchol-5-en-3β-ol BODIPY-cholesterol) [24]. The reader is referred to our previous review paper [6] for critical descriptions of these works and their main conclusions, as here we focus on more recent studies.

We used our 100-ns simulations of NBD-PC in fluid DPPC to calculate the FRET orientation factor (κ^2^) for homo-FRET between those probes embedded in this membrane system [25]. κ^2^ is a measure of the relative orientation of FRET donor and acceptor transition dipoles. Its value is required to calculate the characteristic distance for the FRET interaction, the so-called Förster radius *R*_0_ (see e.g., [26]):

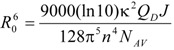
(1)

**Figure 3:**
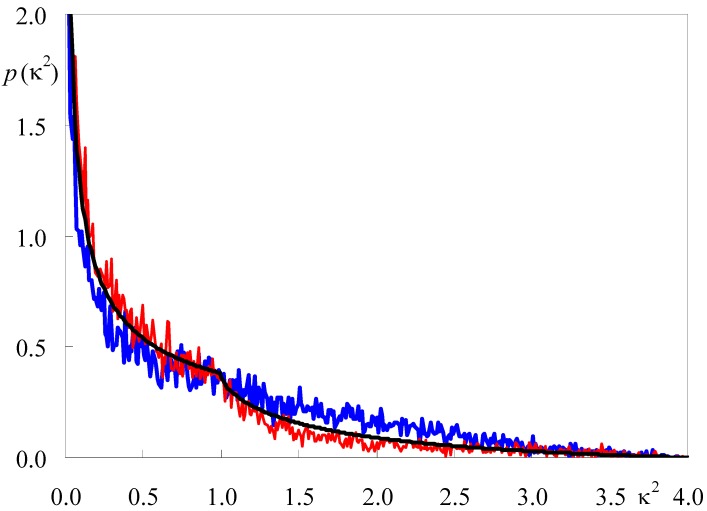
Calculated probability density *p*(κ^2^) for C6-NBD-PC (red) and C12-NBD-PC (blue), compared with the analytical isotropic result of Equation 2 (black). Adapted from [25].


In turn, *R*_0_ is an essential parameter for quantitative description of the kinetics of FRET in membrane systems (e.g., [27,28]), as well as in the classic use of FRET as a spectroscopic ruler [29]. In the preceding equation, *Q_D_* is the quantum yield of the donor in the absence of acceptor molecules, *n* is the refraction index of the medium, *N_AV_* is the Avogadro number, and *J* is the normalized overlap integral between the donor emission and the acceptor absorption spectrum. Whereas *Q_D_* and *J* may be calculated straightforwardly from spectral data, there is no experimental technique suited to a definite measurement of κ^2^ (though it was shown by Dale and coworkers [30] that intervals containing its average value <κ^2^> can be inferred from adequate fluorescence anisotropy measurents). Most often, the theoretical value for the so-called dynamic isotropic limit (<κ^2^> = 2/3) is used, but the <κ^2^> uncertainty is still widely regarded as an inconvenience that may be especially important in membranes, because of their intrinsic anisotropic nature and the restricted rotational mobility experienced by fluorophores incorporated inside the bilayer. However, from the position coordinates in an MD trajectory, calculation of κ^2^ for a given FRET donor-acceptor molecular pair is straightforward, and averaging both over pairs and over time is conveniently carried out. Figure 3 shows the probability densities of κ^2^ for the C6-NBD-PC and C12-NBD-PC systems, compared with the theoretical isotropic orientation result, which is given by Equation 2 (see e.g., [26]).

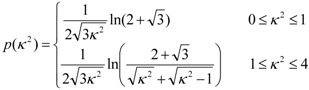
(2)

Although the distributions generally resemble the analytical solutions for isotropic dipoles, <κ^2^> for C12-NBD-PC (0.87 ± 0.06) is ~30% higher than the 2/3 dynamic isotropic limit, whereas <κ^2^> for C12-NBD-PC (0.61 ± 0.03) is slightly lower than that limiting value. These values are discussed in the original reference in terms of preferential orientation of the NBD group approximately parallel to the bilayer plane, as well as some extent of probe aggregation in the case of C6-NBD-PC. To our knowledge, this was the first calculation of κ^2^ for intermolecular FRET by atomistic molecular simulation methods. A previous study, limited to a 100-ps timescale, focused in intramolecular FRET occurring in a non-membrane dye [31]. This methodology provides a way to calculate *R*_0_ with improved accuracy relative to the widespread <κ^2^> = 2/3 assumption, and can also be employed in hetero-FRET κ^2^ calculation, provided that bilayers containing both donor and acceptor probes are simulated.

**Figure 4:**
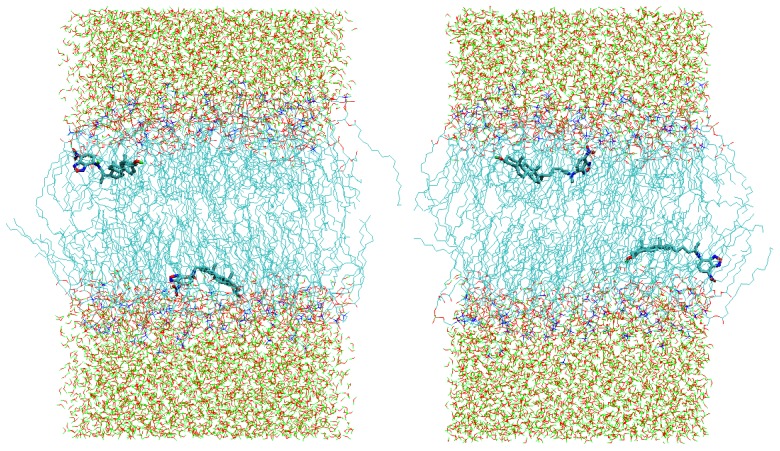
Final snapshots of 100-ns MD simulations of fully hydrated 128-molecule fluid POPC bilayers with either two 22-NBD-cholesterol (left) or two 25-NBD-cholesterol (right) molecules inserted. CH_n_ groups (n = 0–3), O atoms, N atoms and polar H atoms are shown in cyan, red, blue and green, respectively.

Simulations of other NBD membrane probes are currently underway in our laboratory, including the homologous series of fatty amines NBD-C_n_ (see [32] for a recent experimental study of these amphiphiles), the head-labeled phospholipid 1,2-dipalmitoyl-*sn*-glycero-3-phosphoethanolamine-*N*-(7-nitro-2-1,3-benzoxadiazol-4-yl) (NBD-PE) and the NBD-labeled analogs of cholesterol 22-(*N*-(7-nitrobenz-2-oxa-1,3-diazol-4-yl)amino)-23,24-bisnor-5-cholen-3β-ol (22-NBD-Cholesterol) and 25-[*N*-[(7-nitro-2-1,3-benzoxadiazol-4-yl)methyl]amino]-27-norcholesterol (25-NBD-Cholesterol; see Figure 1D and Figure 1E). Concerning the latter, although both 22- and 25-NBD-cholesterol have been employed to study the distribution and dynamics of cholesterol in different systems (for a review, see e.g., [33]), controversy exists regarding the location and orientation of the NBD group of these probes in lipid bilayers. Using time-resolved fluorescence, we showed that 22-NBD-cholesterol prefers the cholesterol-poor liquid disordered (ld) phase to the cholesterol-rich liquid ordered (lo) phase of phase separated PC/cholesterol vesicles [34]. This displacement from the cholesterol-enriched phase is obviously an anomalous behavior for a probe supposed to mimic the behavior of cholesterol. From fluorescence quenching, it was found that the fluorophore of 25-NBD-cholesterol was deeply buried within the bilayer [35]. In contrast, using NMR, it was observed that both NBD-sterols may adopt an upside-down orientation within bilayers [36]. This discrepancy remains unsolved in the literature. MD simulations are well suited to the clarification of this question, and we have very recently simulated both sterols in POPC bilayers (Loura and Ramalho, unpublished data). Although complete trajectory analysis is still underway, it is clear from the structures obtained after 100 ns simulation (Figure 4) that both sterols adopt a conformation where both opposing polar groups (‑OH and ‑NO_2_) are simultaneously oriented towards the interface. This behavior is at odds with the well known orientation of cholesterol, with the hydroxyl group towards the interface, and the long axis approximately perpendicular to the bilayer plane [37,38]. Therefore, this study seems to confirm that both 22- and 25- NBD-cholesterol are inappropriate cholesterol analogs, probably at variance with BODIPY-cholesterol, whose orientation in the bilayer resembles that of cholesterol [24].

Given the significant degree of perturbation induced by phospholipids labeled at the acyl chain with polar fluorophores (as evidenced by the NBD-PC studies described above), it would be reasonable to assume that headgroup-labeled lipid probes present a potentially less membrane-disturbing behavior. This hypothesis can be tested using MD simulations. In this way, two recent reports address specifically the behavior of phospholipids labeled at the headgroup with rhodamine dyes in fluid DPPC bilayers. Skaug *et al.* [39] simulated Texas Red-1,2-dihexadecanoyl-*sn*-glycero-3-phosphoethanolamine (TR-DHPE; Figure 1F), whereas Kyrychenko [40] simulated 1,2-dipalmitoyl-*sn*-glycero-3-phosphoethanolamine-*N*-(lissamine rhodamine B sulfonyl) (Rhod-DPPE; Figure 1G).

**Figure 5:**
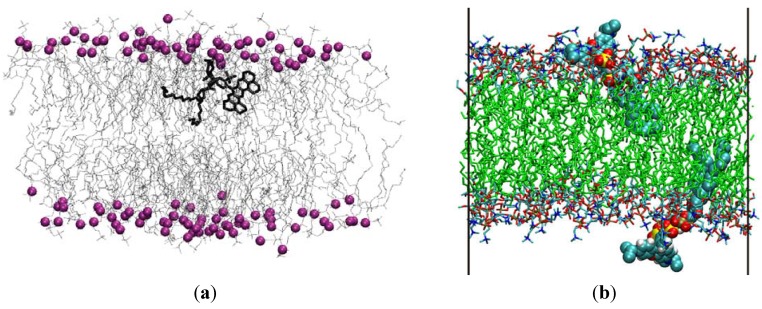
Snapshots of simulations of polar-fluorophore headgroup-labeled phospholipid probes in fluid DPPC bilayers. Water molecules are omitted in both panels. (**a**) 1 TR-DHPE:127 DPPC bilayer [39]. The DPPC phosphorus atoms, purple spheres, are highlighted to show the location of the headgroups. The entire TR-DHPE molecule is shown in thick black. Reprinted with permission from [39]. Copyright 2009 American Chemical Society. (**b**) 2 Rhod-DPPE:126 DPPC bilayer [40]. The Rhod-DPPE molecules are presented in vdW view. Reprinted with permission from [40]. Copyright 2010 Elsevier.

As seen from the structures in Figure 1, these probes share an identical phosphatidylethanolamine lipid moiety, and present quite similar fluorophores attached to the headgroup. In both cases, fluorophore parameterization involved quantum mechanical calculations. For TR-DHPE, density functional theory (at the B3LYP level) was used with a 6-31+G(d, p) basis set to optimize the geometry, and partial charges were assigned using the Merz-Kollman-Singh method [41,42]. For Rhod-DPPE, UB3LYP/6-31G(d)-optimized equilibrium structure and partial electronic charges were used. Although different basis sets were employed in these studies, the similarity of the two fluorophores enables comparison of the partial charges determined in the two studies, shown in Table 1 for selected atoms. It is concluded that higher absolute charges were consistently obtained in the Rhod-DPPE study. Perhaps for this reason, the two studies predict different transverse locations for the two fluorophores at equilibrium, with Texas Red in the upper acyl chain region and lissamine rhodamine B sulfonyl in a more external position, near the water/lipid interface (co-localizing with the phosphate and choline groups of DPPC). This differential behavior, illustrated in the snapshots of Figure 5, is not consistent with parallax analysis of experimental fluorescence quenching results, which predicts identical transverse locations of the two fluorophores in 1,2-dioleoyl-*sn*-glycero-3-phosphocholine vesicles [43], despite TR being slightly more hydrophobic than lissamine rhodamine B sulfonyl. The distinct behaviors of the two simulated probes therefore possibly stem from the differences in the model building procedure, which emphasizes the importance of careful parameterization of these polar fluorophores. It could be argued that the combination of different starting positions and incomplete equilibration would be another possible cause for this difference. In fact, the fluorophore of Rhod-DPPE was located outside the headgroup region (into the water region) in the starting structure [40], whereas that of TR-DHPE appears to have been placed inside the DPPC headgroups [39]. However, both articles report convergence of both average area/lipid and fluorophore transverse position, thus invoking effective equilibration.

**Table 1:** Selected atom charges (see Figure 1F and Figure 1G for numbering) reported for TR-DHPE [39] and Rhod-DPPE [40].

Atom number	1	2	3	4	5	6	7	8	9
**TR-DHPE**	−0.035,	−0.368	0.950	−0.519,	0.979	−0.537,	−0.174	−0.421	1.202
	0.018			−0.591,		−0.558			
				−0.600					
**Rhod-DPPE**	−0.8	−0.6	1.6	−0.8	1.6	−0.7	−0.5	−0.8	1.7

In the study of Skaug *et al.* [39], both 1 TR-DHPE:127 DPPC and 1 TR-DHPE:511 DPPC systems were simulated, with slightly different fluorophore transverse locations and orientations being recovered for the two simulations. As expected from its location, the bulky Texas Red fluorophore introduces local disordering of the DPPC acyl chains apparent from the decrease of –*S*_CD_ (in both systems) and increase in the average area/lipid values (in the 1 TR-DHPE:127 DPPC system only) for DPPC molecules closest to the probe. It was observed that the aryl group of Texas Red and the P atom of a particular DPPC lipid remained in close contact (≅ 0.5 nm of each other) throughout the duration of both 1 TR-DHPE:127 DPPC and 1 TR-DHPE:511 DPPC simulations [39]. On the other hand, the core of the fluorophore of Rhod-DPPE has a tilt of 44° ± 8° relative to the bilayer normal, and 2 Rhod-DPPE:126 DPPC bilayers show reduced –*S*_CD_ (by ~5-10%) values in all positions of the DPPC acyl chains [40]. On the whole, a significantly smaller degree of probe perturbation induced by headgroup-labeled lipid probes, compared to acyl-chain labeled lipid probes, could not be verified, as both studies report disordering effects of probe incorporation on the bilayer structure.

Whereas the above studies concern commercially available fluorescent membrane probes that have found previous use in the membrane fluorescence research community, recent works combine fluorescence measurements and MD simulations to address the behaviour of novel environment-sensitive 2-(2′-Pyridyl)- (Figure 1H) and 2-(2′-Pyrimidyl)-Indoles [44] and 2,6-bis(1*H*-benzimidazol-2-yl)pyridine [45] in POPC. Spectral shifts and variation of steady-state and time-resolved fluorescence in different environments are measured. Several parameters are calculated from the MD simulations, including the kinetics of insertion into the bilayer from the water region, the equilibrium transverse location, the inserted probes’ orientations, hydrogen bonding involving water and probe molecules, and the free energy profile of penetration (estimated using the method of potential of mean constraint force (PMF)). From the latter, free energies of probe insertion are calculated, which agree well with the experimental values obtained from fluorescence intensity variation upon titration with lipid vesicles.

Calculations of the free energy profile of insertion were also carried out for the side chains of fluorescent aromatic aminoacids tryptophan (Trp), tyrosine (Tyr), and phenylalanine (Phe) [46,47,48]. Although these molecules are not strictly fluorescent membrane probes, they are relevant as the fluorescent reporter moieties of membrane proteins. Whereas the side chains of Trp and Tyr have minima of free energy profile near the glycerol region of the bilayer (in accordance with the common interfacial location of Trp residues in membrane proteins’ transmembrane segments), for Phe either an absolute minimum of free energy is observed in the middle of the bilayer [46], or a very flat free energy plateau [48] is recovered for all bilayer locations inside the glycerol region. These studies also addressed the orientation of the ring systems of these compounds. At the center of the membrane, there is a slight tendency for all three residues to align the plane of the ring structure with the lipid tails. This tendency becomes strong in the denser region of the bilayer for Tyr and Trp. For the latter (especially Trp), a tendency to point the polar portion of the side chain toward the membrane interface is observed [48].

## 3. Coarse-Grained Simulations of Dyes in Lipid Bilayers

Coarse-grained models represent molecules in a simplified way, e.g. combining multiple atoms into one interaction site or bead. This allows the simulation of larger systems for longer time than that sampled in atomistic simulations. However, this is achieved at the expense of atomic resolution, which is generally critical to many of the aspects that are commonly studied in MD simulation of fluorophores. One sole coarse-grained study of a fluorescent membrane probe (voltage sensitive dye dibutyl-amino-styryl-pyridinium-butyl-sulfonate or Di-4-ASPBS, see Figure 1I) and its derivatives interacting with lipid (POPC) bilayers has been reported [49], using the MARTINI forcefield [50]. In this work, coarse-grain simulations are used to compute the free energy of binding of these dyes to the bilayer. The determination of this parameter from atomistic simulations is highly demanding from the numerical point of view, and coarse-grained methodologies allow its calculation at a fraction of the computational cost. Validation of the MARTINI simulations is achieved by calculating the fluorophore transverse position and orientation, and comparing them (successfully) with atomistic simulations and experimental data. Constraint options were optimized for the determination of the PMF using umbrella sampling, in order to sample conveniently the water/membrane partition (at the expense of an adequate sampling of flip-flop motions).

Although the study was generally successful, the effects of increasing the lipophilic tail chain length and introducing polar phosphoric acid ester groups at the head or tail of these amphiphiles on the free energy of membrane binding were only semiquantitatively reproduced. Most importantly, the authors noticed an overestimation of the free energy increase with increasing chain length of the Di-4-ASPBS derivatives. The underlying reasons for this were not clear, as the increment in binding free energy was calculated in agreement with experiment for a series of alcohols using the same parameterization and simulation protocols. A possible explanation is that the experimental data might be biased due to dye aggregation in the aqueous phase, especially for the longer-chained derivatives (for which discrepancies are larger). In any case, this study shows that calculation of the partition of membrane-active molecules from careful coarse-grained MD simulations is both fast and sufficiently accurate to constitute a useful tool in computational membrane biophysics.

## 4. Conclusions

The last few years have seen a dramatic increase and diversification of the use of MD simulations to study the behavior of fluorescence membrane probes interacting with lipid bilayers. Examples of studies in all common lamellar lipid phases (most commonly liquid disordered, but also liquid ordered and gel phases) are described. Whereas simple apolar probes such as DPH and pyrene were the first to be studied by these methodologies, recent emphasis has shifted to complex amphiphilic probes, with fluorophores such as NBD, BODIPY, rhodamine or cyanine groups. The number of studies employing this type of probes has clearly surpassed those with the traditional small lipophilic compounds in the last two years. The increase in complexity of the simulated probes has highlighted the importance of adequate parameterization. Methodological differences in parameterization may produce substantially divergent results, as illustrated in the above comparison of the two studies of structurally very similar rhodamine headgroup-labeled phospholipids. For this reason, careful testing against experimentally well-characterized systems is still required. In any case, this review shows that MD simulations constitute a very useful tool in the determination of both probe properties (location, orientation, dynamics) and probe-induced perturbation of the bilayer. Recently, the free energy of probe partition into the bilayer was added to the list of calculated parameters. The determination of the extent of interaction of a given compound with bilayers is usually the first step in the characterization of its behavior in membranes. Unless the amounts of compound in the water and lipid media are known, experimental quantitative characterization of many parameters is not feasible. To this effect, determination of PMF using umbrella sampling is the approach available from MD simulations. Theoretical calculation of the free energy of bilayer partition is particularly advantageous for prediction purposes, as well as in situations where experimental determination is difficult e.g. due to probe aggregation or limited solubility in the aqueous medium. As illustrated by the above described works, although atomistic simulations can be used, coarse-grained simulations provide an attractive option as they allow for much faster computation. For other parameters, atomic resolution is generally essential, and atomistic simulations remain the method of option.
